# Therapeutic efficacy of optimal pulse technology in the treatment of *chalazions*

**DOI:** 10.3389/fmed.2023.1286159

**Published:** 2023-11-21

**Authors:** Xi Song, Chunying Zhang, Saisai Zhang, Mansha He

**Affiliations:** Guangzhou Aier Eye Hospital Affiliated to Jinan University, Guangzhou, Guangdong, China

**Keywords:** *chalazions*, optimal pulse technology, meibomian gland cyst, meibomian gland area, conjunctival congestion

## Abstract

**Introduction:**

To evaluate the efficacy of optimized pulse technology in treating chalazia.

**Methods:**

Prospective before-after study. All patients received two sessions of optimal pulse technology (OPT) with an interval of 1 week. The first visit was before treatment and the patients underwent 2 treatment sessions with a 1-week interval. The non-invasive tear breakup time (NIBUT), corneal fluorescein staining (CFS) score, Schirmer’s test I without anesthesia, conjunctival hyperemia, and meibomian gland area were compared before and after treatment, and the related factors of curative effect were analyzed.

**Results:**

23 patients (23 eyes) with chalazia were included. All patients received two sessions of OPT treatment at 1-week intervals. Following the first OPT treatment, a reduction in the chalazion size was observed in 17 patients (73.91%). One patient was completely cured, and 1 patient had an increase in the diameter of the chalazion. The meibomian gland area increased significantly compared to before treatment (*p* = 0.023). Compared with baseline, the conjunctival congestion and ST decreased, NIBUT increased, and there was no statistical difference. After the second treatment, the chalazion size decreased in 21 cases, and 3 patients were cured. A significant increase in the meibomian gland area compared with the baseline area (*p* < 0.001). Additionally, conjunctival congestion decreased significantly. After two sessions, the Schirmer test exhibited a decrease, and NIBUT increased, although these changes did not reach statistical significance. The curative effect was unrelated to sex, age, first onset, single disease, and other factors.

**Conclusion:**

After treatment, the diameter of chalazions was reduced in 91.3% of the patients, and the area of the meibomian gland was significantly increased compared with that before treatment, which suggested that 2 OPT treatments at an interval of 1 week can improve the signs of adult patients in the non-acute infectious stage with chalazia.

## 1 Introduction

*Chalazion,* a chronic inflammatory granuloma, occurs due to the blockage of meibomian glands and the accumulation of glandular secretions. Among Asians, the estimated prevalence of this condition is almost 4% (1). Despite being a common disease, recurrent episodes and treatments might exacerbate the impairment of the meibomian gland structure and function, resulting in scarring, increased eye discomfort, and decreased quality of life (2). In the presence of an infection, it might develop into orbital cellulitis, posing a potentially life-threatening situation (3).

*Chalazions* typically resolve on their own and can be managed in the initial stages of the disease through the application of a hot compress and conservative drug treatment (4, 5). Curettage, a conventional surgical treatment, is commonly employed for treating *chalazions*. However, certain patients prefer to explore conservative treatment options before considering surgery, possibly due to apprehension regarding the surgical procedure, anesthesia, pain, and other factors. This approach may be particularly favored by individuals experiencing recurrent *chalazions*. Multiple surgical interventions could increase the psychological burden on patients, exacerbate discomfort, and result in skin scars. Additionally, cases of cardio-ocular reflex have been reported associated with using anesthetics (6). Knezevic et al. suggested that multi-site botulinum toxin injections around the cyst safely and effectively treat recurrent *chalazions*. The underlying mechanism of this treatment might be attributed to the ability of botulinum toxin to reduce the meibomian gland’s secretory function and peripheral inflammation through the cholinergic pathway (7). Another alternative to curettage is the local injection of triamcinolone acetonide, as suggested by Amynah et al., which can achieve similar therapeutic outcomes (2, 8). Although the local injection method is simple, severe adverse reactions such as vascular obstruction and vision loss have been reported (9). Therefore, exploring new, non-operative, and safe methods for treating *chalazions* is crucial.

In recent years, the clinical efficacy and safety of intense pulsed light (IPL) for treating ocular surface diseases such as dry eye disease and blepharitis associated with meibomian gland dysfunction (MGD) have been gradually recognized (10–12). Previous retrospective studies have demonstrated that combining IPL with meibomian gland massage could significantly reduce the recurrence rate in patients with recurrent c*halazions* and improve tear film rupture time, meibomian gland secretion function, and other ocular surface signs. Optimal pulse technology (OPT) represents the fifth-generation IPL technology and offers several advantages over the original IPL, including more stable energy delivery and reduced pain (13). However, no prospective study has explored OPT’s efficacy in treating *chalazions*.

**Figure 1:**
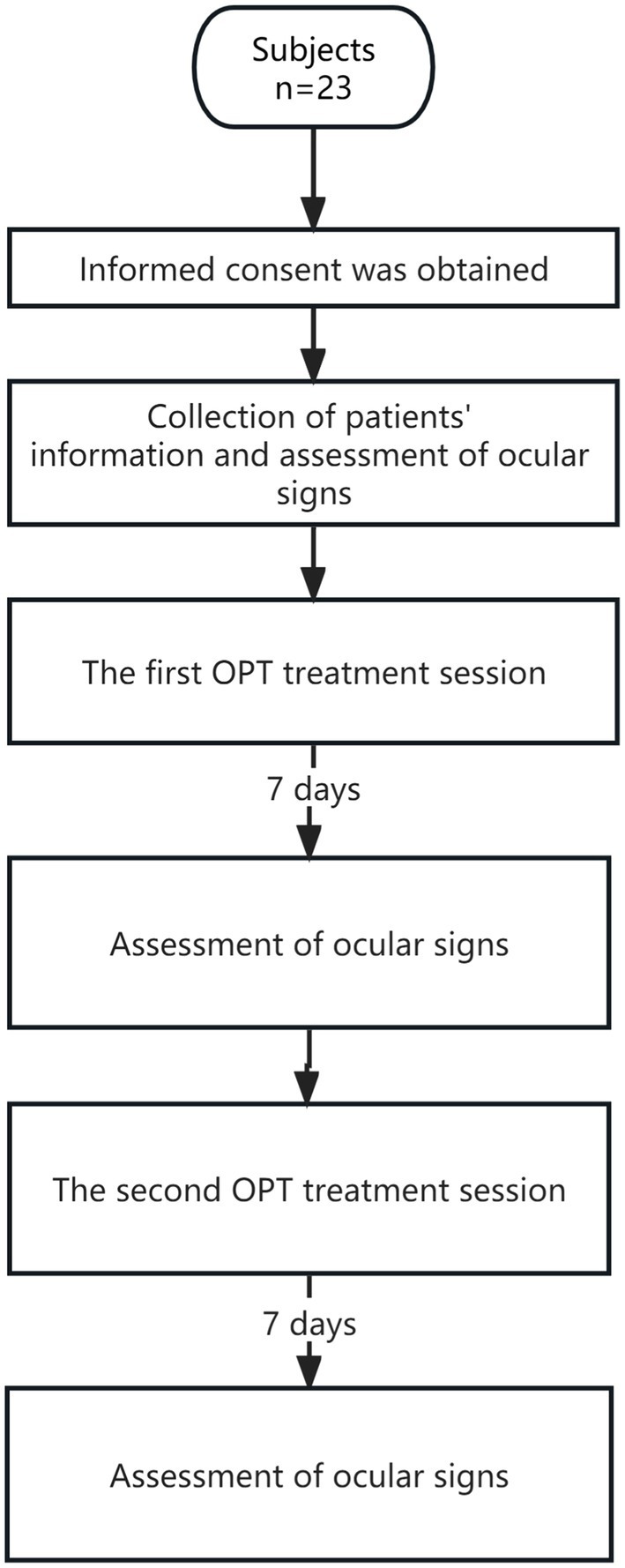
A flowchart of procedures used in this study.

Therefore, this study was initiated to evaluate, for the first time, the efficacy of OPT in treating *chalazions.* This study aimed to explore a clinical, non-invasive approach to managing meibomian gland cysts (Figure 1).

## 2 Methods

### 2.1 Patients

This study adhered to the principles stated in the Declaration of Helsinki and was approved by the Guangzhou Aier Eye Hospital Ethics Committee affiliated with Jinan University (GZAIER2022IRB05). All patients seeking treatment at the outpatient clinic from June 2022 to May 2023 provided signed informed consent. The inclusion criteria for this study were as follows: (1) patients diagnosed with chalazions, including primary and recurrent chalazion; (2) patients aged ≥18 years; (3) those whose eyelid skin and lesion were intact without ulceration and pus; (4) patients in the non-acute infectious stage with chalazion (defined as the secretions from the meibomian glands are released into the tarsus and surrounding soft tissue of the eyelid, leading to an acute inflammatory reaction characterized by pain and erythema) (14). The following criteria were used as contraindications for Optimal pulse technology (OPT) treatment (15): (1) Fitzpatrick skin color classification above type IV; (2) presence of existing eye allergic diseases; (3) abnormal eyelid structure; (4) recent eye or laser treatment within 4 weeks (including cataract surgery, refractive surgery, and fundus laser treatment); (5) Recent tanning or exposure to tanning devices within 4 weeks before treatment; (6) use of exfoliating cosmetics 4 weeks before treatment; (7) a history of skin allergy within 4 weeks before treatment; (8) diagnosis of solar dermatitis, malignant skin tumor or precancerous lesion, solar keratosis, or melanoma; (9) use of medications known to cause photosensitivity; (10) recent oral administration of isotretinoin within 1 month before treatment; (11) the pupil did not return to the standard size after mydriasis; (12) uncured periorbital trauma; (13) patients with neuropsychiatric disorders; (14) patients taking anticoagulant drugs or having coagulation disorders; (15) hypersensitivity to ocular surface drops containing sodium fluorescein or coupling agents; (16) participation in other clinical studies within the first 3 weeks of the study; (17) pregnant or lactating women.

### 2.2 OPT treatment

All patients underwent OPT treatments using M22 (Lumenis Inc., Lumenis, Yokneam, Israel), comprising two sessions with a 7-day interval. The treatment mode employed was OPT, with a wavelength of 590 mm, pulse width of 6.0, 6.0, and 6.0 ms, pulse width delay of 50 and 50 ms, and energy ranging from 10 to 15 J/cm^2^. The treatment procedure was as follows: (1) Patients were informed about the purpose, method, and important considerations of the examination. (2) Patients assumed a supine position, removed their makeup, and cleaned their faces. (3) Corneal contact lenses were placed on the treatment eyes, and an eye shield was inserted. Patients were instructed to close their eyes, and a coupling agent (1–2 mm) was evenly applied to the upper and lower eyelid skin. (4) The light guide head delivered energy from the outer canthus to the inner canthus sequentially, ensuring that the spot coverage did not overlap or repeat. Intensive treatment was performed in areas above, below, nasal, temporal, and central to the meibomian gland cyst. This process was repeated twice, resulting in 20 treatment points per eyelid. A 3-s pause was observed before each treatment to allow sufficient skin cooling. (5) After treatment, the eye shield and contact lens were removed. (6) The patient’s skin reaction and pain during treatment were observe The patient’s skin reaction and pain during treatment were observed. Mild or moderate redness of the treatment site skin and mild tingling sensation experienced by the patient were considered normal reactions. However, if the skin exhibited severe redness and swelling, and the patient could not tolerate the pain, the energy was reduced, or the treatment was stopped. Adverse reactions at the treatment site were monitored after the treatment. Patients were advised to apply sunscreen and moisturize their skin within 48 h after treatment and avoid washing their face with hot water. The patients were followed up before each treatment and 7 days after the completion of the treatment course. Throughout the treatment process, the patients were instructed to use hot compresses and antibiotic eye drops (0.5% Levofloxacin eye drop, Japan). In cases where the volume of chalazia did not decrease by more than 80% following the second treatment session, surgical intervention was considered.

### 2.3 Other inspection methods and data collection

The diameter of the chalazion decreased by 80% was defined as adequate. The following parameters were measured to evaluate the treatment efficacy: non-invasive tear breakup time (NIBUT), corneal fluorescein staining (CFS) score, Schirmer’s test I without anesthesia, conjunctival congestion and meibomian gland area. The CFS score was evaluated based on grades 0 to 3 in each quadrant, resulting in a total score according to the National Eye Institute’s grading scale (16). The conjunctival congestion score was evaluated based on grades 0 to 3 in temporal and nasal bulbar conjunctiva using the OCULUS (Keratograph, Oculus Company, Germany). The meibomian gland area was evaluated by capturing photographs of the patient’s upper and lower eyelid glands using OCULUS. The images were then analyzed using Image J software to calculate the ratio of the meibomian gland area to the eyelid area. The eye with a more significant number of chalazions was used for analysis. The right eye was chosen for analysis in cases where the eyes were comparable.

### 2.4 Statistical method

The data obtained in this study were analyzed using SPSS version 27.0 and plotted using Prism 8.0. Descriptive statistics for normally distributed data are presented as the mean ± standard deviation (SD), while median and quartile range was used for non-normally distributed quantitative data (IQR). Conjunctival congestion, fTBUT, meibomian gland area, and ST values before and after each treatment session were compared using the paired *t*-test, whereas CFS scores before and after treatment were compared using the Wilcoxon rank sum test. The chi-square test was used for between-group comparisons. Binary Logistics regression analysis were used to analyze the factors associated with the effectiveness of OPT treatment. *p*-values < 0.05 were considered statistically significant.

## 3 Results

23 patients (23 eyes), including 4 males and 19 females, were enrolled in the study. The average age of the patients was 31.61 ± 11.42 years old. The patient demographics were shown in Table 1. The patients did not have a specialist’s diagnosis of any systemic diseases, such as diabetes, hypertension, or hyperlipidemia.

**Table 1:** Baseline characteristics of chalazion patients enrolled in the study.

Characteristics	*n* = 23, *n*(%)
Age (years), mean ± SD (range)	31.61 ± 11.42(18–59)
<45 years	20(87.0%)
≥45 years	3(13.0%)
**Gender, *n* (%)**	
Male	4(17.4%)
Female	19(82.6%)
**Laterality, *n* (%)**	
Unilateral chalazions	18(78.3%)
Bilateral chalazions	5(21.7%)
**First onset, *n* (%)**	
Primary chalazions	17(73.9%)
Recurrent chalazions	6(26.1%)
**Quantity, *n* (%)**	
Single	18(78.3%)
Multiple	5(21.7%)
**Diameter of the chalazions, *n* (%)**	
≤5 mm	21(91.3%)
>5 mm	2(8.7%)
**Position, *n* (%)**	
Upper eyelid	14(60.9%)
Lower eyelid	9(39.1%)

Among them, 3 patients were older than 45 years old, 20 were younger than 45 years old, 18 had unilateral disease, 5 had bilateral chalazions, 17 were the primary chalazions, 18 had only one chalazion in one eyelid, and 2 patients had a diameter of chalazion more significant than 5 mm.

Following the first OPT treatment, a reduction in the *chalazion size was observed* in 17 patients (73.91%), increased in 1 patient (4.35%), and remained unchanged in 5 patients (21.74%; Table 2). The meibomian gland area was significantly increased from 43.6 ± 2.87 to 45.97 ± 3.18 (*p* = 0.023). Compared with baseline, the conjunctival hyperemia was reduced from 0.99 ± 0.36 to 0.94 ± 0.28, ST was reduced from 8.94 ± 6.01 to 8.69 ± 7.45, and NIBUT was increased from 8.18 ± 4.46 to 9.53 ± 4.21, although these changes did not reach statistical significance (Table 3).

**Table 2:** The number of patients whose diameter of chalazions decreased, increased, or remained unchanged after OPT treatment.

	7 days after first session		7 days after second session	
	Reduction(*n*)	Increased or unchanged(*n*)	Reduction(*n*)	Increased or unchanged(*n*)
**Age**				
<45 years	16	4	19	1
≥45 years	1	2	2	1
*P*-value	0.155		0.249	
**Gender**				
Male	4	0	4	0
Female	13	6	17	2
*P*-value	0.539		1.000	
**Episodes**				
Primary	12	5	16	1
Recurrent	5	1	5	1
*P*-value	1.000		0.462	
**Quantity**				
Solitary	12	5	15	2
Multiple	5	1	6	0
*P*-value	1.000		1.000	
**Diameter of chalazions**				
≤5 mm	16	5	20	1
>5 mm	1	1	1	1
*P*-value	0.462		0.170	
**Position**				
Upper eyelid	12	2	13	1
Lower eyelid	5	4	8	1
*P*-value	0.162		1.000	

**Table 3:** Comparison of ocular signs between baseline and 7 days after first and second treatment of OPT sessions.

	Baseline	After one session	After two sessions	*p*-value (baseline vs. T1)	*p*-value (baseline vs. T2)	*p*-value (T1 vs. T2)
Conjunctival congestion	0.99(0.36)	0.94(0.28)	0.81(0.29)	0.177	**<0.001** ^***^	**0.014** ^*^
ST (mm)	8.94(6.01)	8.69(7.45)	9.13(9.14)	0.124	0.083	0.960
NIBUT (s)	8.18(4.46)	9.53(4.21)	9.71(4.18)	0.680	0.760	0.856
CFS	0.09(0)	0.04(0)	0.00(0)	0.157	0.317	0.564
Meibomian gland area (%)	43.69(2.87)	45.97(3.18)	50.92(2.45)	**0.023** ^*^	**<0.001** ^***^	**0.004** ^**^

CFS, corneal fluorescein staining; NIBUT, non-invasive tear breakup time; ST, Schirmer’s test I; T1, 7 days after first treatment; T2, 7 days after second treatment. *p < 0.05, **p < 0.01, ***p < 0.001. The values in bold were used to remind the reader that these *p* -values were statistically significant.

**Figure 2:**
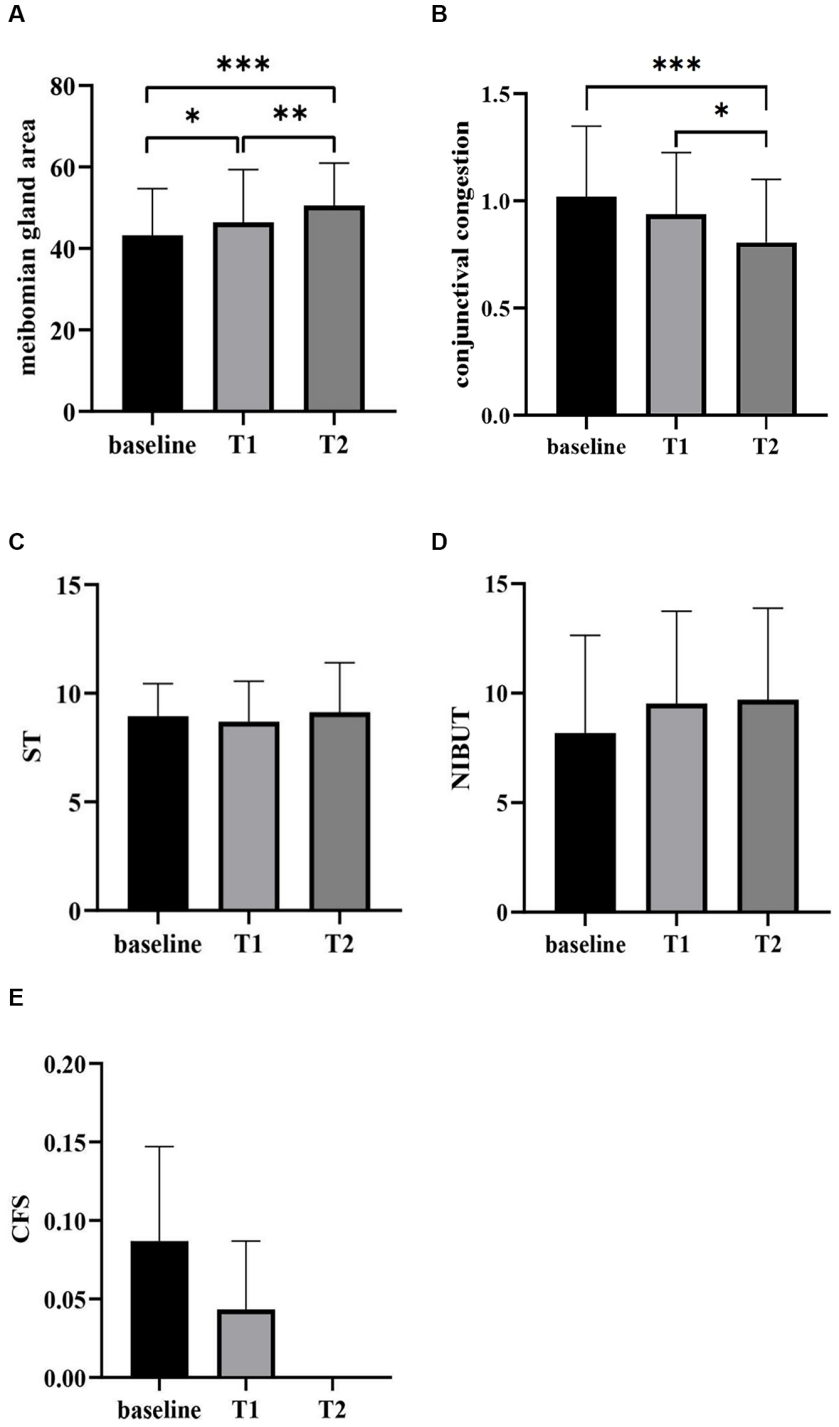
Comparison of the meibomian gland area **(A)**, conjunctival congestion **(B)**, ST **(C)**, NIBUT **(D)** and CFS **(E)** before and after OPT treatment sessions. T1, 7 days after the first treatment; T2, 7 days after the second treatment; ^*^p < 0.05, ^**^p < 0.01, ^***^p < 0.001.

Following the second OPT treatment, the number of patients with reduced meibomian gland cyst diameter grew to 21 (91.3%), 3 of which were completely cured. In 2 cases, the size of the *chalazion* remained unchanged (8.7%). The patient with enlargement of the chalazion after the first treatment had a reduction in chalazion size after the second treatment, indicating improvement (Table 2). Compared with baseline, the meibomian gland area was significantly improved to 50.92 ± 2.45 (*p* < 0.001), and conjunctival hyperemia was significantly decreased to 0.81 ± 0.29 (*p* < 0.001) after the second treatment (Figure 2). There were no significant differences in the NIBUT and ST at the first and second treatments (*p* > 0.05; Table 3). The CFS score was zero before, after one treatment, and after two sessions.

Age over 45 years old, gender, first onset, single lesion, diameter of meibomian gland cyst before treatment, and lesion located in the upper or lower eyelid had no relationship with the reduction of meibomian gland cyst diameter (*p* > 0.05; Table 2).

The addition of the meibomian gland area was not associated with age over 45 years, gender, first onset, single lesion, diameter of meibomian gland cyst less than 5 mm before treatment, and lesion located in the upper or lower eyelid (*p* > 0.05; Table 4).

**Table 4:** The number of patients whose meibomian gland area decreased, increased, or remained unchanged after OPT treatment.

	7 days after first session		7 days after second session	
	Increased(*n*)	Reduction or unchanged(*n*)	Increased(*n*)	Reduction or unchanged(*n*)
**Age**				
<45 years	14	6	18	2
≥45 years	3	0	2	1
*P*-value	0.539		0.365	
**Gender**				
Male	2	2	3	1
Female	15	4	17	2
*P*-value	0.270		0.453	
**Episodes**				
Primary	12	5	14	3
Recurrent	5	1	6	0
*P*-value	1.000		0.539	
**Quantity**				
Solitary	13	4	14	3
Multiple	4	2	6	0
*P*-value	0.632		0.539	
**Diameter of chalazions**				
≤5 mm	15	6	18	3
>5 mm	2	0	2	0
*P*-value	0.379		0.567	
**Position**				
Upper eyelid	11	3	13	1
Lower eyelid	6	3	7	2
*P*-value	0.643		0.538	

The adequacy of the OPT treatment was not correlated with sex, age, first onset, the diameter of the chalazion before treatment, upper eyelid or lower eyelid location, or the presence of a single or multiple chalazions according to our research (*p* > 0.05).

During the treatment and follow-up, 9 patients underwent hot compress, 21 patients had received antibiotic eye drops, and a total of 2 patients underwent surgical intervention after two sessions of OPT treatment. No adverse reactions, such as skin pigmentation, skin rupture, corneal epithelial defect, uveitis, and cataract were observed during the treatment and follow-up.

## 4 Discussion

Chalazia is a common eye condition. While the pathogenesis of MGD and blepharitis concerning chalazions remains unclear, previous studies have indicated a strong association (1, 17). When the regular discharge of oil from the meibomian gland is disrupted, excessive meibum accumulates in the surrounding eyelid tissue, resulting in inflammatory cell infiltration, redness, and swelling (18). Over time, this process can result in cyst formation. The treatment approach for chalazion in the non-acute infection stage typically involves warm compress and administration of antibiotic eye drops. Jacob Evans indicated that surgical intervention was required in approximately 40% of patients following the administration of topical antibiotic and steroid therapy (1). IPL is a broad-spectrum pulsed light with a wavelength of 500–2,000 nm, which is a non-invasive and non-laser treatment method. Initially, IPL was primarily used for treating rosacea. However, in 2002, its application expanded to include the treatment of dry eyes, meibomian gland dysfunction, blepharitis, and other ocular surface diseases. Currently, OPT and intense regulatory pulsed light (IRPL) are the main IPL devices in clinical practice. The OPT is the latest generation (fifth-generation) IPL technology, offering enhanced stable energy and reduced discomfort compared with the original IPL (19). Compared with IRPL technology, OPT technology has the advantages of no pulse spike, constant energy, stable and repeatable, larger flash density, and a more stable cooling system (20). Subsequent studies have increasingly demonstrated the safety and efficacy of IPL and OPT in improving symptoms and signs in patients. Although the exact mechanism of how OPT treats ocular surface diseases remains to be elucidated, it is believed that OPT can inhibit the release of inflammatory mediators within the local tissues and impede the inflammatory cascade reaction (21). In this study, conjunctival congestion improved significantly after two OPT treatments, which supports the aforementioned hypothesis. By generating heat that can penetrate the skin, OPT selectively targets the meibomian gland, resulting in the melting of eyelid esters and an improvement in the palpebral ester character (22, 23). Consequently, this process reduces the cyst diameter and increases the area of the meibomian gland. In our study, a significant improvement was observed in the meibomian gland area of the patients who underwent OPT treatment. Furthermore, as the number of treatments increased, the meibomian gland area increased more significantly, indicating that OPT exhibits a persistent and cumulative effect when targeting the meibomian gland. Moreover, OPT used hemoglobin as its target color base. During treatment, light is absorbed by hemoglobin, converting it into heat. This localized increase in temperature results in the solidification and sealing of abnormally dilated capillaries along the palpebral margin. This process not only helps reduce bacteria and mites present on the surface of the eyelid skin but also contributes to the overall efficacy of the treatment.

IPL energy penetrates the eyelid, gets absorbed by hemoglobin, and transforms into heat, causing localized destruction of superficial blood vessels. This process reduces eyelid neovascularization and subsequently lowers the accumulation of inflammatory mediators, thereby eliminating a significant source of inflammation in both the eyelid and meibomian glands. Simultaneously, the volume of meibomian gland cysts diminishes post-treatment, leading to a reduction in the levels of inflammatory factors, which may have accounted for the observed improvement in conjunctival hyperemia following treatment. In Arita’s research, the therapeutic effects of IPL on refractive multiple recurrent chalazia were examined, it was noted that corneal fluorescein staining (CFS) significantly improved in patients with recurrent meibomian gland cysts after OPT treatment, Schirmer’s Test (ST) did not exhibit statistically significant changes, and non-invasive tear breakup time (NIBUT) alterations were not assessed in their study (24). In addition, a study by Yirui Zhu focusing on patients with chalazion in the acute inflammatory phase, reported significant improvements in NIBUT after OPT treatment (25). These outcomes contrast with the patient population in our current study. In our study, although no statistically significant differences were observed in NIBUT and CFS after one or two OPT treatments compared to baseline, NIBUT displayed an upward trend, suggesting improved tear film stability. Similarly, CFS exhibited a downward trend, indicating reduced ocular surface staining. ST showed a decrease after the first treatment, which we attribute to factors such as the short evaluation interval and the OPT treatment effect not yet reaching its full potential. Interestingly, ST increased from baseline after the second treatment. Our study encompassed a diverse range of chalazion types, including patients in non-acute infectious stages, as well as primary and recurrent cases, with varying diameters and locations of chalazion. Following two treatments, NIBUT, CFS, and ST all demonstrated an improving trend compared to their respective baseline measurements.

**Figure 3:**
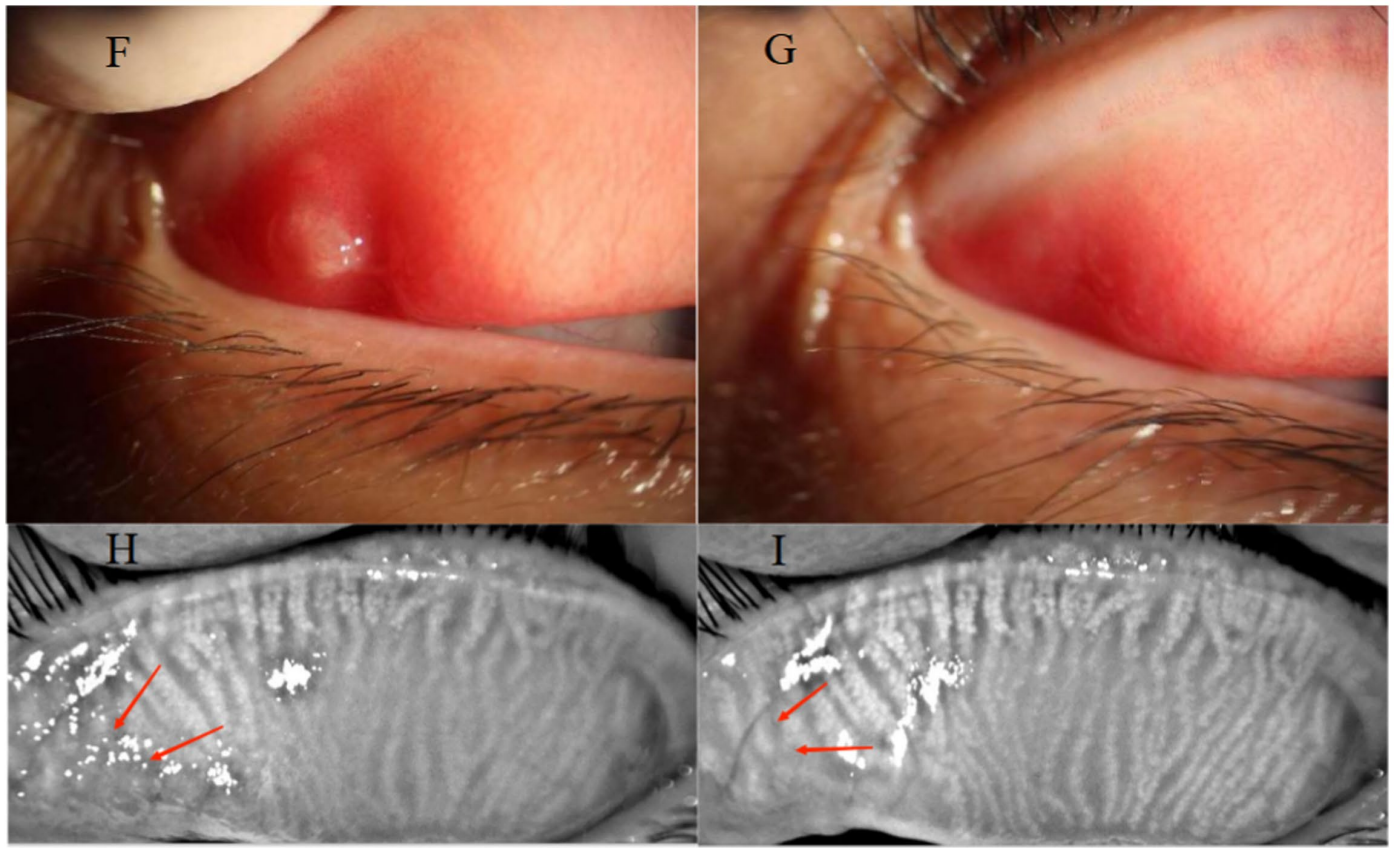
A 32-year-old female presented with a primary chalazion in the upper eyelid of the right eye (F). Before OPT treatments, the patient showed palpebral conjunctival hyperemia (F) and meibomian gland dropout at the site of the cyst (H). Following treatment, complete regression of the cyst was observed (G), along with decreased hyperemia on the palpebral conjunctival (G) and increased of the meibomian gland area at the cyst site (I).

The frequency of OPT treatment is also a critical aspect to investigate. Previous studies have indicated that the effects of OPT are cumulative and persistent. Jin et al. conducted a study suggesting that four sessions of OPT combined with meibomian gland expression can effectively alleviate symptoms, reduce palpebral margin congestion, improve meibomian gland secretion function, and increase tear film rupture time in patients with MGD (23). Furthermore, the improvement becomes more evident with an increase in the number of treatment sessions, and there is still a statistically significant difference observed 3 months after completing the treatment course. In the Chinese experts’ consensus on IPL for treating MGD-related dry eyes, it is recommended to undergo IPL treatments 2–12 times (15). Reiko et al. have suggested that patients with recurrent chalazions might require three or more courses of IPL treatment (24, 25). Given the specific characteristics of the patients enrolled in our study, which included individuals with single, small-diameter, and primary chalazions, and considering the aforementioned recommendations regarding the number of IPL treatments, a total of two OPT treatments were administered (Figure 3). During the follow-up, it was found that one patient recovered after 1 week of treatment. The patient was a 30-year-old woman who underwent chalazion curettage 6 months before our intervention. The diameter of her chalazion was 2 mm before treatment. Notably, the patient remained free from relapse during the irregular follow-up assessments conducted after the treatment. Furthermore, three additional patients were completely cured after undergoing two treatments. These positive outcomes have left the patients delighted with the rapid onset of OPT efficacy and the level of comfort they experienced.

The effectiveness of performing two OPT treatments for treating chalazions has demonstrated its clinical relevance.

As previous studies have indicated, *chalazions* are commonly observed in adult women, particularly around the age of 25 years. In our analysis of related factors influencing OPT efficacy while treating *chalazions,* no significant associations were observed with sex, age, first onset, the presence of a single chalazion, site, and the diameter of the meibomian gland cysts. These findings are consistent with previous studies.

This study has certain limitations. First, the sample size was small, the observation period was short, and more objective and microscopic observations of the expression of inflammatory factors in ocular surface tissues, such as optical surface goblet cells, epithelial cells, and tears, were lacking. These aspects warrant further investigation and will be our focus in future research endeavors.

## 5 Conclusion

In our study, the primary chalazion was observed in 73.9% patients, while cysts larger than 5 mm in diameter were found in 8.7%, additionally, single cysts were present in 78.3% patients. After treatment, the diameter of chalazions was reduced in 91.3% of the patients, and the area of the meibomian gland was significantly increased compared with that before treatment. To the best of our knowledge, this is the first study to explore the efficacy of OPT treatment on different types of chalazions, which suggested that two OPT treatments at an interval of 1 week can improve the signs of adult patients in the non-acute infectious stage with chalazion.

## Data availability statement

The original contributions presented in the study are included in the article/Supplementary material, further inquiries can be directed to the corresponding author.

## Ethics statement

The studies involving humans were approved by Guangzhou Aier Eye Hospital Ethics Committee affiliated with Jinan University. The studies were conducted in accordance with the local legislation and institutional requirements. The participants provided their written informed consent to participate in this study.

## Author contributions

XS: Funding acquisition, Methodology, Resources, Writing – original draft, Writing – review & editing. CZ: Formal analysis, Investigation, Writing – review & editing. SZ: Formal analysis, Investigation, Writing – review & editing. MH: Conceptualization, Supervision, Writing – review & editing.
