# Supplementary material for: Therapeutic efficacy of optimal pulse technology in the treatment of chalazions
Source: Front Med (Lausanne). 2023 Nov 21;10:1286159. doi: 10.3389/fmed.2023.1286159 (PMC10702728; doi:10.3389/fmed.2023.1286159)
Supplement: Supplementary file 1 [file Table_1.DOC]

**Supplementary Table 5.** Topical and surgical treatments during OPT treatments and follow-up

| Interventions | n=23 (%) |
| --- | --- |
| Topical treatment for chalazions during OPT treatment, n (%) |  |
| Warm compresses | 9(39.1%) |
| Antibiotic eye drops(0.5%Levofloxacin Eye Drops) | 21 (91.3%) |
| Underwent surgery after OPT treatment | 2(8.7%) |
